# Anatomical connections underlying personally-familiar face processing

**DOI:** 10.1371/journal.pone.0222087

**Published:** 2019-09-11

**Authors:** Daylín Góngora, Ana Maria Castro-Laguardia, Johanna Pérez, Pedro Valdés-Sosa, Maria A. Bobes

**Affiliations:** 1 The Clinical Hospital of Chengdu Brain Science Institute, MOE Key Lab for Neuroinformation, University of Electronic Science and Technology of China, Chengdu, Sichuan, China; 2 Department of Cognitive Neuroscience, Cuban Neuroscience Center, Havana, Havana, Cuba; 3 Department of Neuroinformatic, Cuban Neuroscience Center, Havana, Havana, Cuba; Nathan S Kline Institute, UNITED STATES

## Abstract

Familiar face processing involves face specific regions (the core face system) as well as other non-specific areas related to processing of person-related information (the extended face system). The connections between core and extended face system areas must be critical for face recognition. Some studies have explored the connectivity pattern of unfamiliar face responding area, but none have explored those areas related to face familiarity processing in the extended system. To study these connections, diffusion weighted imaging with probabilistic tractography was used to estimate the white-matter pathways between core and extended system regions, which were defined from functional magnetic resonance imaging responses to personally-familiar faces. Strong white matter connections were found between occipitotemporal face areas (OFA/FFA) with superior temporal sulcus and insula suggesting the possible existence of direct anatomical connections from face-specific areas to frontal nodes that could underlay the processing of emotional information associated to familiar faces.

## Introduction

Face recognition is crucial for social interactions and many studies have tried to understand the neural system underlying familiar face processing. These studies revealed some regions in the occipitotemporal brain areas preferentially responding to faces and they have been repeatedly identified using functional magnetic resonance imaging (fMRI), revealing the “core” of face processing [1–4]. The “core” regions are the fusiform face area (FFA), the occipital face area (OFA) and the superior temporal sulcus (STS). These areas are more responsive to faces than to any other type of stimuli such as objects or scenes (especially in the right hemisphere) [5]. The classical model of neural basis of face perception [6] attached to this core unit a so-called extended system, comprising regions from neural systems involved in other cognitive functions that can be recruited to act in concert with the regions in the core system to extract meaning from faces [1, 6, 7]. Some of these areas can be in charge of retrieving semantic information and affective response, such as the hippocampus [8], the insula [9] and the amygdala [2, 10–12]. The activation of these brain regions by faces has been replicated in many studies using different tasks [1, 6, 13, 14].

Functional connectivity studies have suggested that the core system is hierarchically organized in a predominantly feed-forward fashion and the processing of emotional expression is based on the coupling between the fusiform gyrus and the amygdala, whereas identity recognition is based on the coupling between the fusiform gyrus and the orbitofrontal cortex [11]. However, the anatomical route underlying these couplings between the core and extended system is not well understood yet.

Some studies have used deterministic tractography and regions of interest (ROIs) defined *a priori* to explore the connections of the core processing system toward frontal areas [15–17]. These studies have delimited the main white matter tracts between occipitotemporal nodes and either temporal or frontal lobes [18]. These tracts are known as inferior longitudinal fasciculus (ILF) and inferior fronto-occipital fasciculus (IFOF). The IFOF connects inferior-lateral and dorsolateral frontal cortex with posterior temporal cortex [19, 20] and continues posteriorly the occipital lobe [21]. The ILF passes along the lateral wall of the occipital and temporal horns of the lateral ventricle [21] and its fibers project in the superior, middle, and inferior temporal and fusiform gyri and also to the lingula, cuneus, the lateral surface of the occipital lobe and the occipital pole [19, 22].

However, the deterministic methods cannot allow the study of connectivity patterns between gray matter nodes (where the fractional anisotropy is low), being the probabilistic methods the proper frame to analyze the circuitry among functionally determined areas [23]. These probabilistic methods initiate a high number of possible paths from every seed, then an index is assigned to the ‘paths’ to express how likely they are to represent the actual path of the nervous fibers. This process is repeated several times with a new set of directions each time [24].

The anatomical gray matter-gray matter connections of the core face areas have been previously examined in a small number of studies [3, 25] by seeding the tractography in fMRI defined face regions of interest of the core system (FFA, OFA, STS). These studies defined ROIs from fMRI localizer by contrasting unfamiliar faces versus non-face conditions and described the connections among the core face system regions. They found no evidence for direct anatomical connections between FFA and STS, contrary to predictions based on current cognitive models; but they did find direct connections to the amygdala from early occipital areas which might underlie a rapid recruitment of limbic brain areas by visual inputs bypassing more elaborated extrastriate cortical processing [25]. They also found no evidence of direct connections to the frontal lobes, where some information contained in familiar faces is processed. These results suggest a complicated pattern of anatomical connections inside the face processing network.

Even when these studies have provided important information about connectivity in the core system, this information is incomplete, since they all defined functional face areas using only unfamiliar faces. It is well-known that face recognition involves not only the initial face analysis carried out by the core areas, but also the analysis of additional information associated to familiar faces, which involves the coupling between core face areas (e.g. the fusiform gyrus) and other structures of the extended face system located in temporal and frontal areas [11]. It has been demonstrated that FFA is a complex area with multiple face selective loci, that can be revealed with different face conditions, specially different for familiar and unfamiliar conditions [26]. Therefore, it is important to delimit precisely the gray matter nodes involved in familiarity face processing and examine all possible connections among them.

In this study, probabilistic tractography was used to explore the anatomical connectivity underlying the links between core and extended system nodes during familiar face processing. Familiar face processing regions were localized by recording fMRI BOLD (Blood-oxygen-level-dependent) response during the presentation of familiar faces (tailored to each participant) and unfamiliar faces as well as non-faces stimuli. This methodology allows the localizing of functional nodes responding to familiar faces including those belonging to the core and extended systems. All these nodes, including the insula (Ins), anterior cingulate and medial orbitofrontal cortex (mOF/AC), medial cingulate (MC) and posterior cingulate (PC), as well as OFA/FFA and posterior STS (pSTS), were used as seeds for the tractography and the probability of connection among all of them. The aim of this study is to explore in more detail the anatomical connectivity patterns between the core and extended system of face processing, including all functional activated areas during familiar face processing.

## Materials and methods

### Sample

The sample used in the fMRI study included 16 individuals of 26 ± 1.9 years old (6 females) with no neurological or psychiatric history. All participants were native Spanish speakers and 11 of them were right-handed (ascertained by self-report). Nine of the 16 subjects participating in functional magnetic resonance scanning, took part of the diffusion tensor imaging scanning protocol (26 ± 1.5 years old, two left-handed subjects, 3 females). These experimental procedures were previously approved by the Ethics Committee of the Cuban Neuroscience Center and are in line with the principles stated in the declaration of Helsinki (1996) [27]. Besides, other nine subjects aged 27 ± 5.7 years old (right-handed subjects, 5 females) were scanned to increase the diffusion weighted sample and the experimental protocol was approved by the local Ethical Committee at University of Electronic Science and Technology of China, in compliance with the latest revision of the Declaration of Helsinki. The totality of the subjects was recruited voluntarily specifically for the purposes of this study. All subjects gave written informed consent.

A dataset of 18 subjects from Human Connectome (HCP) project, specifically the Young Adult dataset [28], was used for validation of our results (https://ida.loni.usc.edu/login.jsp). The HCP project is the result of efforts of co-investigators from the University of Southern California, Martinos Center for Biomedical Imaging at Massachusetts General Hospital (MGH), Washington University, and the University of Minnesota (Principal Investigators: Bruce Rosen, M.D., Ph.D., Martinos Center at Massachusetts General Hospital; Arthur W. Toga, Ph.D., University of Southern California, Van J. Weeden, MD, Martinos Center at Massachusetts General Hospital) and has been supported by the National Institute of Dental and Craniofacial Research (NIDCR), the National Institute of Mental Health (NIMH) and the National Institute of Neurological Disorders and Stroke (NINDS).

### Magnetic resonance imaging acquisition protocol

The data from the first sample was recorded in a Siemens 3.0T Magnetom Allegra, while the second sample was recorded using 3.0T General Electric Discovery MR750 system. The scanning protocol included, for each subject, a high resolution T1-weighted anatomical image and a standard diffusion sequence.

In the Siemens scan the T1-weighted structural image (1 x 1 x 1 mm resolution) was acquired for further coregistration with the following parameters: echo time (TE) = 3930 ms, repetition time (TR) = 1940 ms, flip angle = 9° and field of view (FOV) = 256 x 256 with 176 contiguous 1 mm-thick slices in a sagittal orientation. Also, the diffusion-weighted images (DWI) were acquired along 80 independent directions. The scan protocol set 75 slices spaced at 2 mm, with 2 mm x 2 mm in-plane resolution and a diffusion weighting b-value of 1000 s/mm^2^. Seven reference images (b0 images) with no diffusion weighting were also obtained (b = 0 s/mm^2^). The following parameters were used: FOV = 128 x 128, TE = 83 ms, TR = 9400 ms, flip angle = 90°.

Using the General Electric system, the T1-weighted structural image (1 x 1 x 1 mm resolution) was registered with the following parameters: TE = 2984 ms, TR = 6884 ms, flip angle = 9° and FOV = 256 x 256 with 172 contiguous 1 mm-thick slices in a sagittal orientation. The DWI was acquired along 80 independent directions as well, and the volumes were conformed of 75 slices spaced at 2 mm, with 2 mm x 2 mm in-plane resolution. The diffusion weighting b-value was 1000 s/mm^2^ and four b0 images were also obtained. The following parameters were used: FOV = 128 x 128, TE = 6240 ms, TR = 8500 ms, flip angle = 90°.

The functional sequence data were collected in the first sample on the Siemens scanner using a T2*-weighted gradient-echo, echo-planar imaging sequence (EPI) with TR = 3000 ms, TE = 1800 ms and flip angle = 90°. The number of acquired slices was 47 with a slice thickness of 2.0 mm. For each run of the experiment 208 volumes were acquired. The first five volumes were discarded from the analysis due to the T1 saturation effect.

### Stimulation paradigm

The stimulation paradigm consisted in an event-related design. Stimuli from three different experimental conditions: familiar faces, unfamiliar faces and houses‚ were randomly presented. Scrambled faces were also included as catch trials. Ten different items were included for each experimental condition. They were repeated six times on each of the two runs of the experiment. Trials of scrambled faces appeared 20 times in each run and subjects were required to press a key every time they saw it.

Stimuli were presented with a duration of 1000 ms and a variable inter-stimuli interval between 4000 and 6000 ms. All of them were projected onto the center of a black screen and viewed through a mirror fixed to the head coil. They consisted on digitalized black and white photographs, equalized in luminance and size. Familiar faces stimuli were tailored to each participant, consisting in frontal pictures from their family members or close acquaintances (boyfriend/girlfriend, mother, father or closest friends) which were masked getting rid of external attributes, except for the face and hair. The unknown faces were balanced in age and gender with familiar faces and all of them presented neutral expression. Participants performed two runs with a duration of 11 min duration each.

### fMRI processing

Preprocessing was carried out with SPM8 (Wellcome Department of Imaging Neuroscience, London, UK) (http://www.fil.ion.ucl.ac.uk/spm) [29]. The functional scans were first submitted to artifact correction using the ArtRepair toolbox (http://cibsr.stanford.edu/tools/ArtRepair/ArtRepair.htm/) thus repairing motion signal intensity outliers and other artifacts (including interpolation using nearest neighbors for bad scans). Then they were submitted to slice scan-time correction and afterward correction for head movements (and extraction of motion parameters) and unwarping. Each mean preprocessed functional image was then coregistered with the subject's T1 image. Spatial normalization was carried out after estimating the normalization parameters from the segmentation of the anatomical T1 in every subject. Finally, all functional images were spatially smoothed using a Gaussian kernel with FWHM of 8 x 8 x 8 mm.

Individual subject data for each run were then analyzed by using a general linear model (GLM) deconvolution with separate regressors for each experimental condition, to estimate the hemodynamic response. The GLM parameters were estimated using the weighted minimum mean square that corrects the temporal correlation in the data [30].

First level and random-effect group analysis were performed resulting in the obtention of functional responsive areas from the core system directly from the t-contrast defined by the contrast unfamiliar faces > houses what evinced face-selective areas (p < 0.001 uncorrected). The extended system regions were obtained from the contrast familiar faces > unfamiliar faces which uncover those areas related to familiarity processing (p-value < 0.01 uncorrected).

### Region of Interest (ROI)

First level and the second-level analysis allowed the definition of functional activated areas related to the evaluated task for single subject and group. For the core system, functional ROIs were obtained directly from the t-contrast defined as unfamiliar faces > houses what evinced face-selective areas (p<0.05 uncorrected). The extended system ROIs were obtained from the contrast familiar faces > unfamiliar faces which uncover those areas related to familiarity processing (p-value<0.05 uncorrected). This relaxed p-value was used to avoid the dilatation of the ROIs into the white matter to maximize contact with streamlines during tractography [3, 25]. The MarsBaR toolbox [31] (http://marsbar.sourceforge.net/) was used for defining the functional ROIs from these previous contrasts. The resulting regions from the contrasts unfamiliar faces > houses were OFA/FFA (left and right) and STSp (left and right) representing core system, while familiar faces > unfamiliar faces uncovered the mOF/AC, MC, PC, left and right Ins, belonging to the extended system of face processing. The ROIs were then transformed to each individual brain in diffusion space automatically, using a programmed routine in MATLAB 2014a [32] using SPM toolbox functionalities [29]. In this routine the high-resolution anatomical T1 image was realigned to the standard position on the AC-PC plane and normalized using the procedures of SPM. The unnormalized T1 was rigidly co-registered with the b0 image using a mutual information cost function [33]. The functional ROIs obtained from the second-level analysis underwent an affine transformation into the original T1 space and were transformed into the Montreal Neurological Institute (MNI) space using the warping parameters estimated from the T1 image. The normalized functional ROIs were then transformed back to the b0 image native brain space of each individual automatically via inverse normalization matrix. For the validation data from HCP a split of OFA and FFA was performed based on the overlapping over the Automated Anatomical Labeling Atlas (AAL) [34].

### DWI processing

The diffusion images obtained were motion and eddy-currents corrected using FSL package (http://www.fmrib.ox.ac.uk/fsl/fdt/index.html) [35–37]. The resulting rotations were used to realign the gradient directions matrix. Non-brain tissue was removed from DWI using Brain Extraction Tool (BET-FSL) [38] with a fractional intensity threshold of 0.3. After that, *bedpostx* function based on Markov Chain Monte Carlo sampling built up distributions on diffusion parameters at each voxel [23, 39]. This procedure creates all the files necessary for running probabilistic tractography.

The probabilistic tractography was performed using *probtrackx2* function which repetitively samples from the distributions of voxel-wise principal diffusion directions, each time computing a streamline through these local samples to generate a probabilistic streamline or a sample from the distribution on the location of the true streamline [23, 39]. The multiple masks option was chosen to generate a connectivity distribution among the ten seed masks extracted from the fMRI analysis with 5000 fiber iterations in each voxel and a threshold curvature angle of 80 degrees, step size of 0.5 mm and a minimum length threshold of 0 mm. That means that the fiber tracking was initiated in both directions (from seed to target and vice versa). The final connectivity paths were summarized by averaging across the subjects and the results were represented using MRIcroGL (http://www.cabiatl.com/mricrogl/).

### Analysis

Since two DWI samples were used, an independent sample permutation t-test (*mult_comp_perm_t2*) was performed between the probabilistic tractography output of both samples to ensure they can be considered as samples of the same population [40, 41]. For this analysis each group was normalized by its estimated standard deviation and the difference between groups was taken. Multiple comparison correction was also applied.

A t-test for dependent samples was performed to uncover the hemispheric asymmetry in connectivity strength among ROIs extracted from fMRI using a p-value = 0.0016 (equivalent to p-value = 0.05 Bonferroni correction), in terms of size or number of voxels. For this analysis was used the STATISTICA 10 package [42]. In this analysis each ROI was considered (as seed) with their ipsilateral connections (regions in the same hemisphere used as target). Besides, the density of visits in each structure of the AAL atlas [34] was estimate and the results submitted to a t-test to explore the hemispheric connectivity strength in the 58 structures contained in each hemisphere. The hemispheric asymmetry was also assessed in 18 subjects from the HCP database using the same processing pipeline and ROIs extracted from the second-level analysis fMRI. This issue was also evaluated using the tracking among individual ROIs (from the first-level analysis of fMRI) in the 9 subjects that were fMRI recorded. The probability path across all seeds from second-level analysis was normalized and averaged and the result represented over the MNI template and the same procedure was done for individual ROIs from first-level analysis on each individual brain from the fMRI-recorded subjects. Besides, probability maps based on subject coincidence were created taking the voxels with more than 5000 visits for each subsample from the two scanners used in this study.

The connectivity matrixes obtained from the probabilistic tractography for each ROI were averaged across the subjects from our sample and validation dataset from HCP, using the aforementioned ROIs and independent OFA and FFA, respectively. Besides, graphical representation of the connectivity was performed by using CircularGraph toolbox from MATLAB (https://www.mathworks.com/matlabcentral/fileexchange/48576-circulargraph/). The line width in the circular graph depicts the normalized sum of count of visits of each pair or ROIs in both directions (using both ROIs of each pair once as seed and once as target). To show the strength of connectivity we used histograms that show the number of count of visits in each ROI normalized by the maximum across all subjects.

## Results

**Fig 1:**
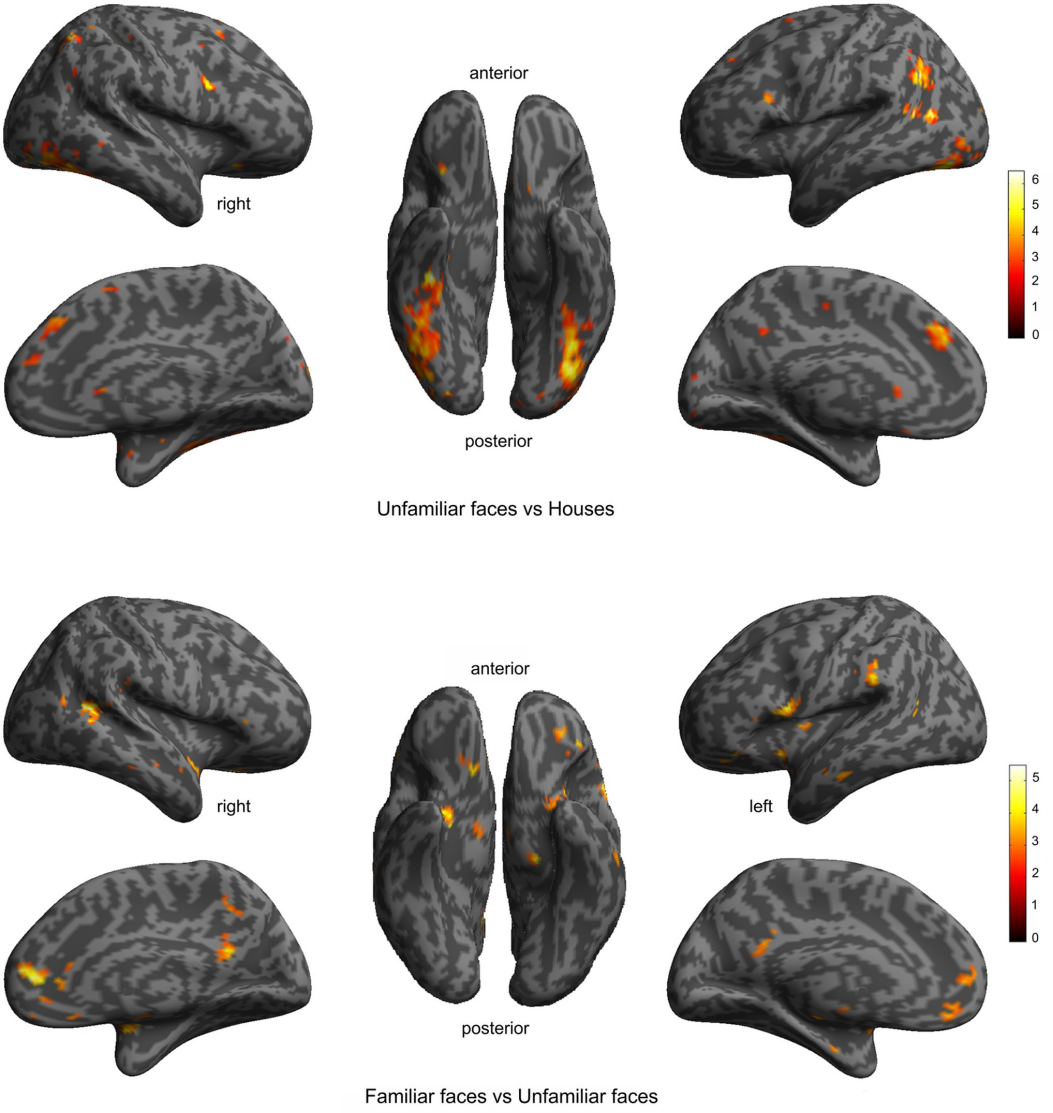
Functional activation identified by the contrasts: A) faces > houses and B) familiar faces > unknown faces. Maps were overlaid on the average inflated cortical surface of the SPM. The figure depicts voxels surviving p< 0.001 and p < 0.01 uncorrected, respectively. Color indicates the t-values.

The fMRI analysis evinced those functional areas related to face processing (Fig 1). The results of the unfamiliar faces > houses contrast identified a big cluster in bilateral occipitotemporal lobe including OFA and FFA (OFA/FFA), left posterior superior temporal sulcus (STSp_L), right posterior superior temporal sulcus (STSp_R) and also small clusters in more occipital areas (visual cortex (Vis)) (Fig 1A). On the other hand, a total of five responsive areas were obtained from the contrast familiar faces > unfamiliar faces evinced clusters in several brain regions including medial orbitofrontal/anterior cingulate (mOF/AC), medial cingulate (MC), posterior cingulate (PC) and left and right insula (Ins_L, Ins_R) (Fig 1B).

**Fig 2:**
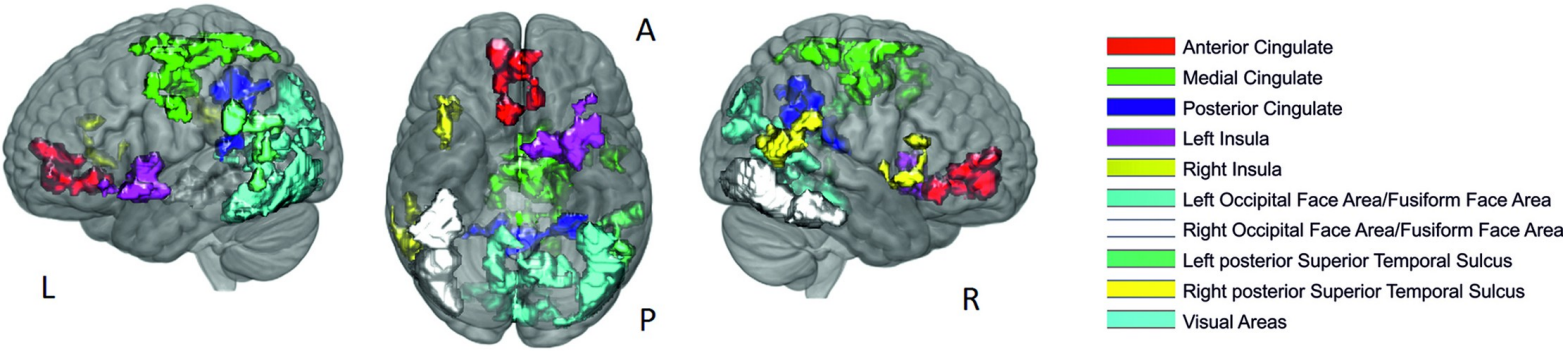
Regions of interest extracted from activation clusters in left and right view (L and R) and ventral view (A: anterior and P: posterior region).

All the identified ROIs (Fig 2) extracted from these activation clusters were used later to performed probabilistic tractography among them using each one as seed. The size of these ROIs in normalized space is listed in Table 1.

**Table 1:** Description of the region of interest (ROI) size obtained from activation clusters.

Regions of Interest (ROIs)	Number of Voxels (normalized space)
Medial orbitofrontal/Anterior cingulate (mOF/AC)	902 (554 in right hemisphere)
Medial cingulate (MC)	2774 (310 in right hemisphere)
Posterior cingulate (PC)	1195 (545 in right hemisphere)
Left insula (Ins-L)	773
Right insula (Ins-R)	204
Left Occipital face area/Face fusiform area (OFA/FFA-L)	1406
Right Occipital face area/Face fusiform area (OFA/FFA-R)	2171
Left Posterior Superior Temporal Sulcus (STSp-L)	730
Right Posterior Superior Temporal Sulcus (STSp-R)	736

The activation clusters obtained in the core system for face processing were larger in the right hemisphere, as well as mOF/AC from the extended system. On the contrary, the rest of the areas, that also belong to the extended system were more represented in the number of voxels over the left hemisphere (MC, PC, Ins) (Table 1).

The two samples of this study were merged after the independent sample permutation t-test showed no significant difference between the probabilistic tractography output of both samples. This result allowed the analysis of all the data altogether as part of the same population.

**Fig 3:**
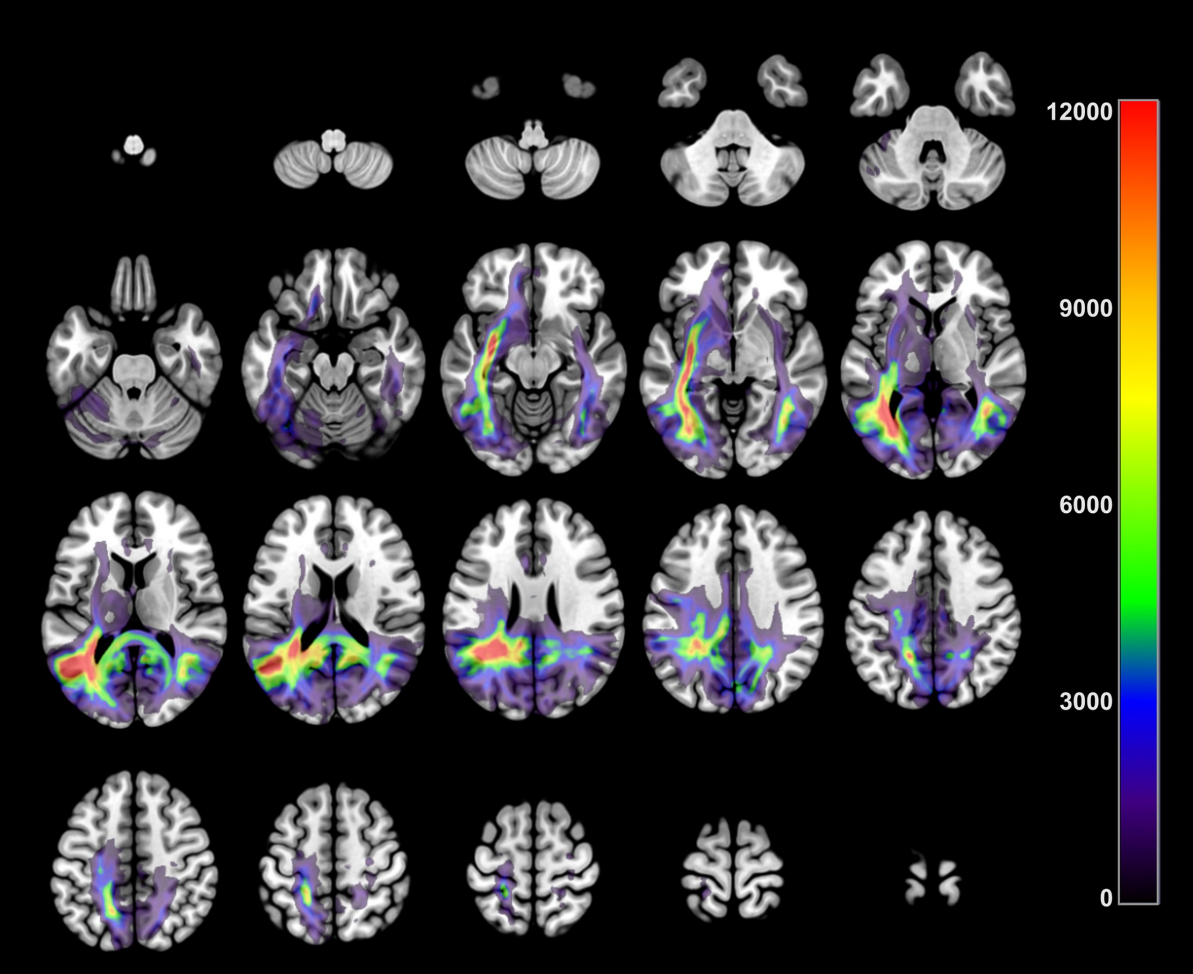
Connectivity path among the functional seeds obtained using probabilistic tractography. The average of count of visits is drawn in each voxel. Color indicates the number of visits.

The reconstruction of the averaged probabilistic path is shown in (Fig 3) indicating a high probability of connections in occipitotemporal areas (core system) but maintaining a stronger pattern towards more frontal areas (extended system) in the left hemisphere. This pattern remained even when the path obtained from the samples recorded in each scanner are represented separately in a subject coincidence map (S1 Fig).

To explore the existence of asymmetry in connectivity strength, we performed a t-test for dependent samples to the count of visits between both hemispheres. For this, each ROI was considered (as seed) with their ipsilateral connections (regions in the same hemisphere used as target). This analysis showed that core areas (OFA/FFA and STSp) were more bidirectionally connected in the left hemisphere while STSp also was more connected to the left representation of Ins (Table 2).

**Table 2:** T-test for dependent samples exploring the lateralization of the probabilistic path among bilateral regions of interest (ROIs) as seeds and the ipsilateral targets (p<0.0016).

Seed Region of Interest	Target Region of Interest	t	P
OFA/FFA Left vs Right	STSp Left vs Right	7.3396	0.000001
STSp Left vs Right	OFA/FFA Left vs Right	7.80803	0.000001
STSp Left vs Right	Ins Left vs Right	3.80462	0.001417

The comparison among density of count of visits in parcellations coming from the AAL atlas showed a left asymmetry in 33.62% of the structures and 0.86% to the right in our main data, while in HCP data the left hemisphere obtained the highest values in the 32.75% of the structures and 19.83% towards the right hemisphere. However, when the analysis was performed using the individual ROIs in the 9 subjects with fMRI recording no difference between hemisphere was founded (S2 Fig). For statistical details refers to Table in S1 Table.

**Fig 4:**
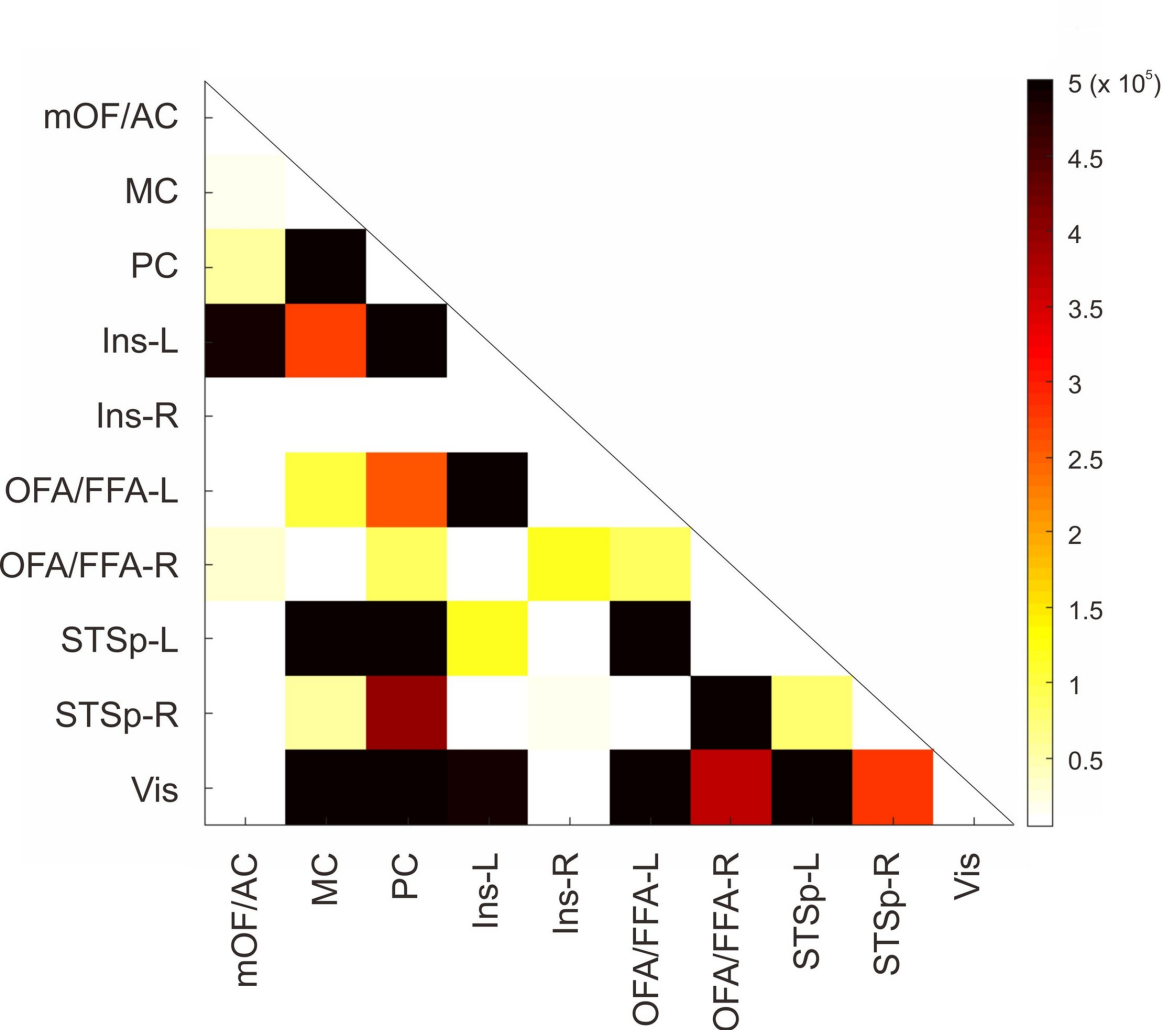
Averaged connectivity matrix across the subjects of cumulated count of visits in each ROI.

**Fig 5:**
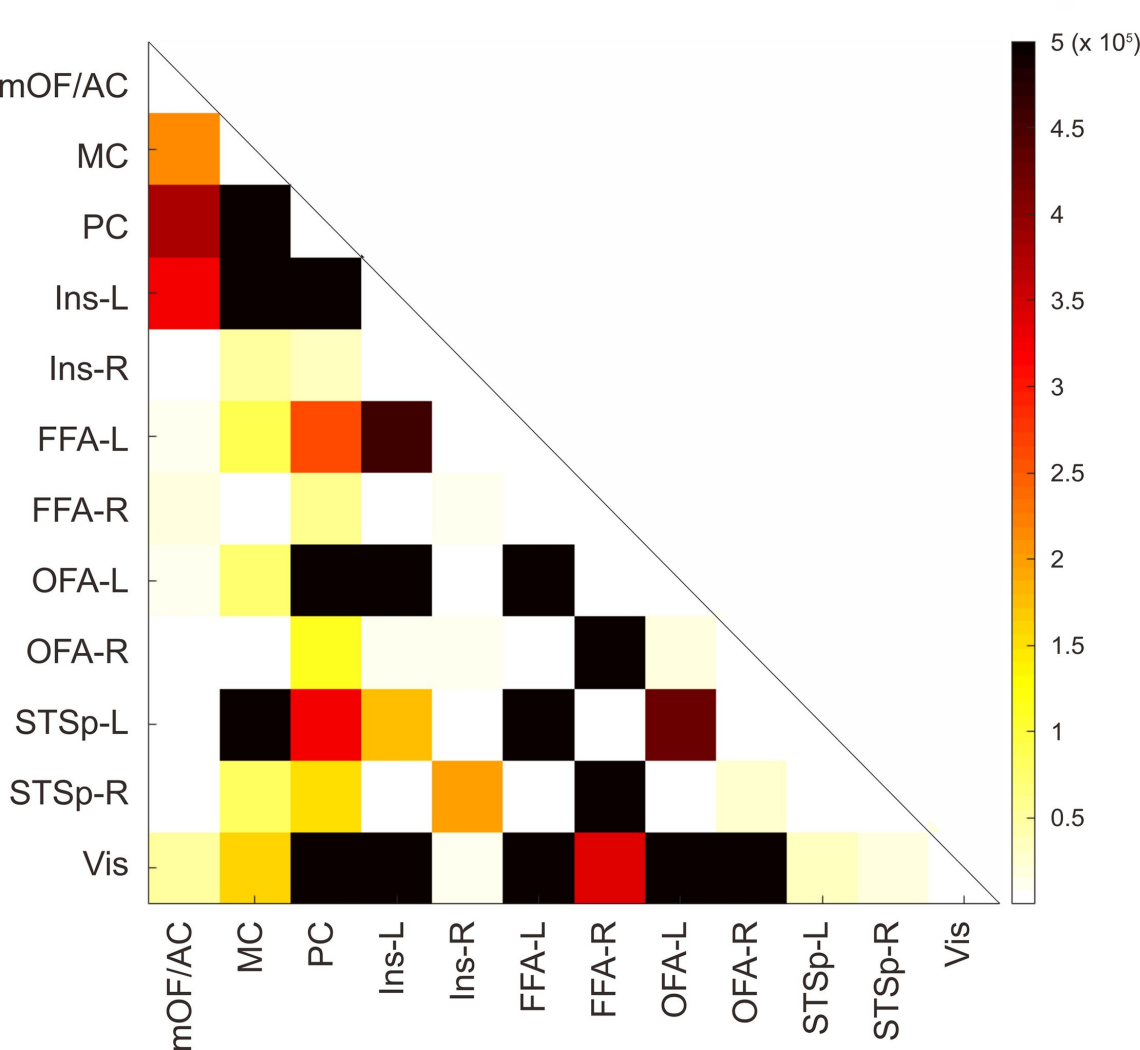
Averaged connectivity matrix across the sample from human connectome project´s subjects of cumulated count of visits in each ROI and considering Occipital Face Area (OFA) and Fusiform Face Area (FFA) as independent seeds.

The average of the count of visits in each ROI was summarized in a typical connectivity matrix (Fig 4) showing the specific areas or ROIs that are strongly connected. The MC was preferably connected to PC but also with left STSp, while PC presented reciprocal connections to MC but also to left STSp (and these ones with PC and MC). The left insula was connected with its corresponding hemispheric OFA/FFA and PC (bidirectionally). Bilateral OFA/FFA was highly connected to its ipsilateral STSp, reciprocally. The Vis was connected mostly to MC, PC, left OFA/FFA and left STSp. Even though our interest was focused in the main structures (OFA and FFA) of the core system as seed of our tractography as a whole, we also estimated the averaged connectivity matrix from 18 subjects from Human Connectome Project by using independent OFA and FFA which can be seen on Fig 5.

**Fig 6:**
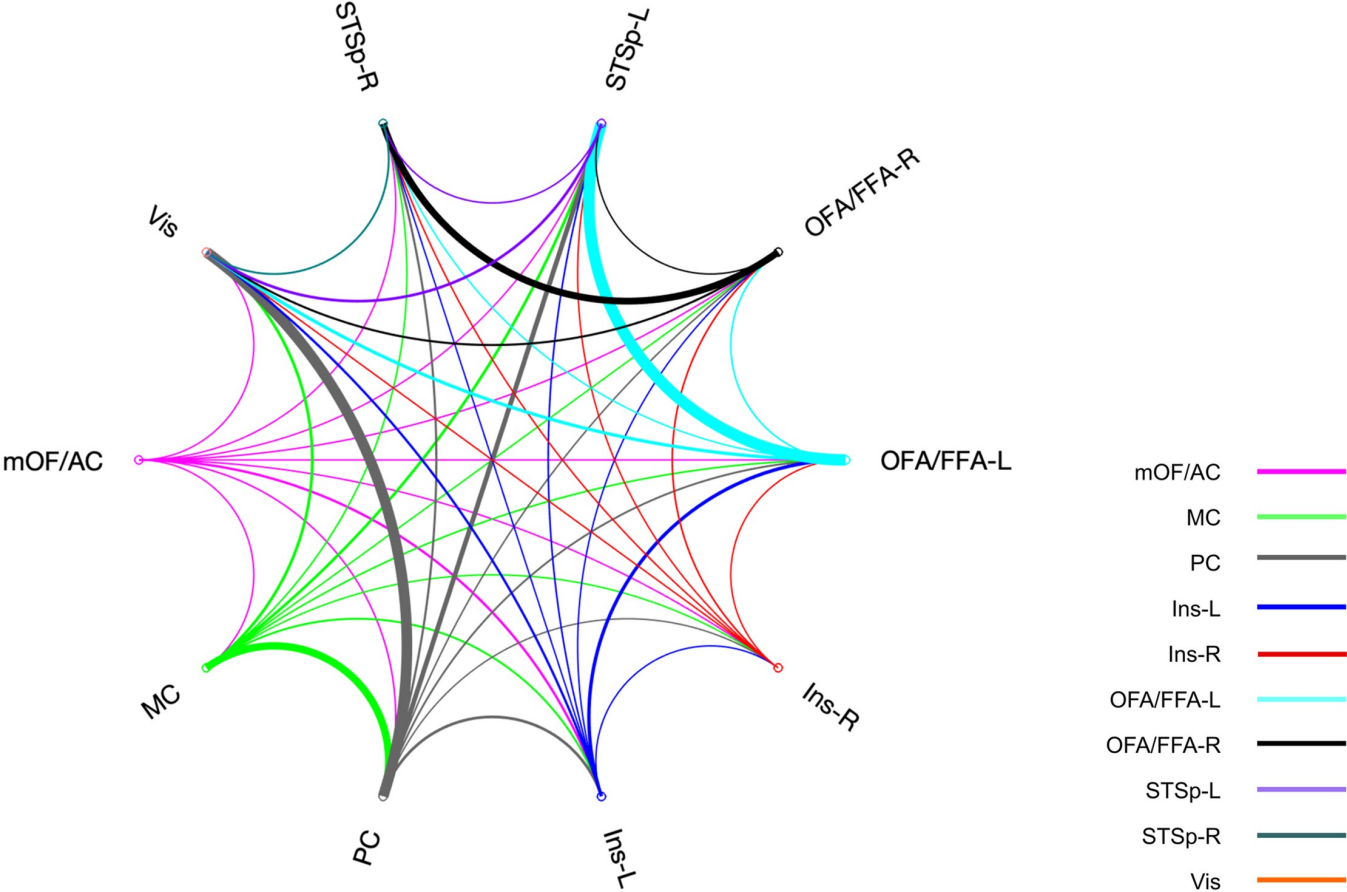
Circular graph of the sum of count of visits from and to each ROI. Line width is normalized to the maximum number of count of visits.

A circular graph was reconstructed from the connectivity matrix among the regions of interest and we pointed out the interesting connections by the edge width (Fig 6). The edge width represents the normalized sum of count of visits of each pair of ROIs but in both functions: each pair once as seed and once as target. As can be seen, the circular graph shows that OFA/FFA (left and right) is preferentially connected to the ipsilateral representation of STSp, in both hemispheres but stronger in the left hemisphere (Fig 6). Besides, left OFA/FFA is well-connected to left Ins despite of the distance between them. The PC appears to be highly connected to Vis and the MC.

**Fig 7:**
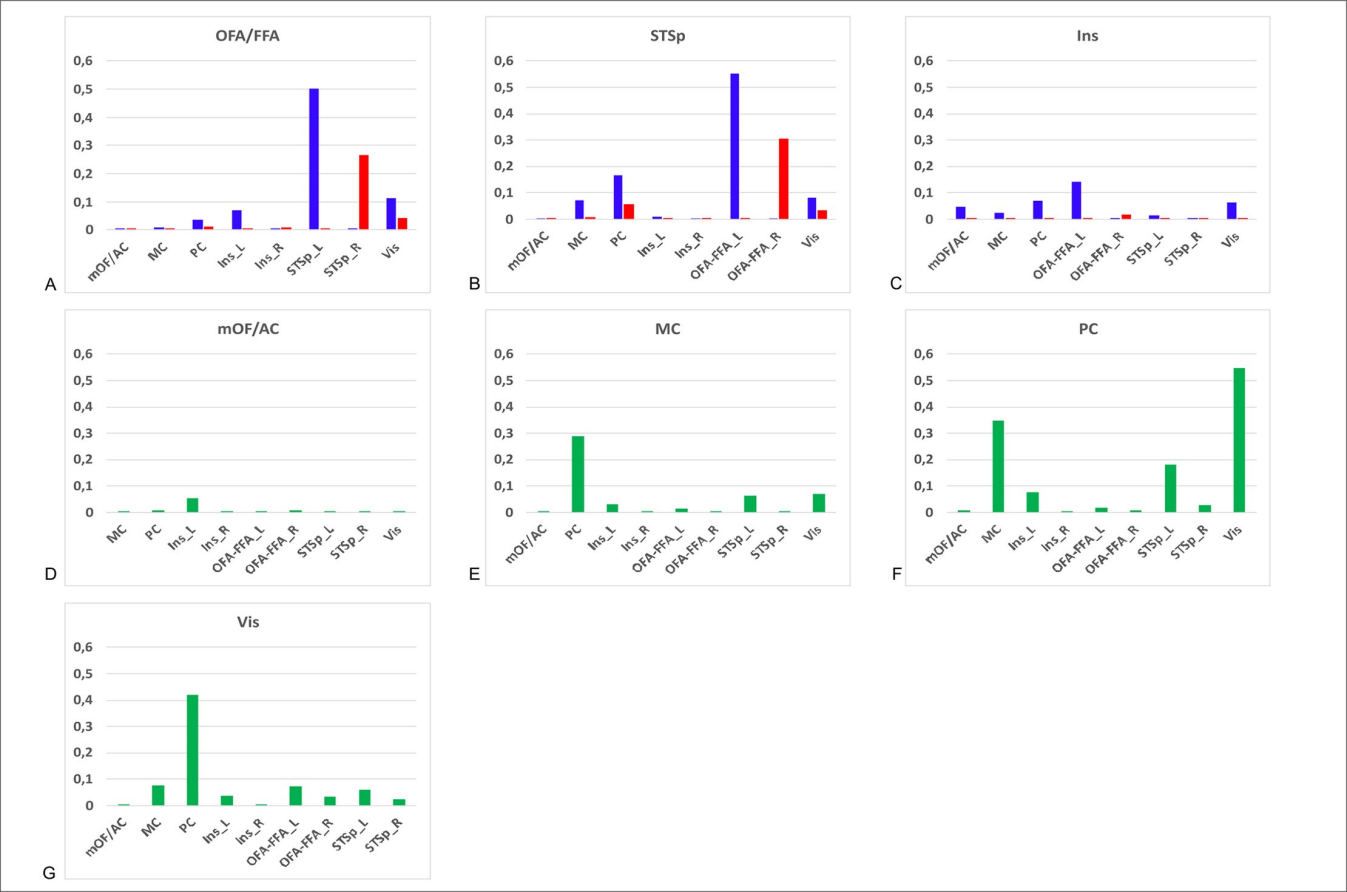
Connectivity probability among regions of interest (ROI) that belong to face processing system. Histograms represent the normalized number of streamlines leaving each seed region towards the target regions. Core system areas: A) Occipital face area-Fusiform face area (OFA/FFA) and B) posterior Superior Temporal Sulcus (STSp); Extended system areas: C) Insula (Ins), D) medial Orbito-frontal cortex/Anterior cingulate (mOF/AC), E) medial cingulate (MC), F) posterior cingulate (PC) and G) Visual cortex (Vis). L: Left hemisphere (blue), R: right hemisphere (red).

These results are corroborated by the histogram of connection probability that represents the normalized number of count of visits leaving each ROI (as seed) (by the maximum across all the subjects) toward the other ones (as target) (Fig 7). Histograms show that OFA/FFA exhibits strong connectivity patterns toward their ipsilateral STSp and Vis (Fig 7A), while there is a noticeable left asymmetry. Meanwhile, the STSp was connected mostly to PC, Vis and ipsilateral OFA/FFA with higher values in left connections (Fig 7B). For the extended system, more subtle connectivity patterns were extracted. The Ins was connected in low degree to different areas but the left one had always the highest values of connections and preferentially to left OFA/FFA (Fig 7C). The mOF/AC showed weak connections with all the regions (Fig 7D); MC was relatively highly connected to PC (Fig 7E). Meanwhile, PC was widely connected to MC, left STSp and Vis (Fig 7F). The Vis was in general poorly connected popping up only its connection to PC (Fig 7G).

## Discussion

The aim of this study was to describe the anatomical connections underlying personally familiar face processing which has not been done before. To directly investigate the structural connections related to face processing task, a probabilistic fiber tracking analysis was conducted using the activation clusters as seeds and targets. In general, the connectivity was higher among ROIs that belong to the same hemisphere, which has been previously reported by Zalesky et al. (2010). Zalesky et al. using diffusion weighted imaging tractography and the 82-node AAL parcellation reconstructed an adjacency matrix that if ordered in such a way that all left-hemisphere nodes occupy the first 41 rows, the presence of two strongly connected sub-blocks along the diagonal become obvious, which exclusively corresponds to intra-hemispheric connectivity [43]. Interestingly, we found direct connections between OFA/FFA, STSp and Ins, which were stronger among the regions in the left hemisphere (Fig 3). This result suggests the existence of multiple parallel pathways from core face areas through extended system.

Considering that probabilistic tractography estimates a probability of connection between the seed voxel and other voxels or regions of the brain (targets), and the probability of connection is usually defined as the density of the trajectories that connect them (where the density is calculated as the number of connecting trajectories normalized by the total number of trajectories) [44], the more voxels in seed and/or target the higher the probability of connections between them. Taking this into account, it is possible that left lateralization in OFA/FFA-Ins connections (and the reciprocal pair, Ins-OFA/FFA) are explained by the larger size of left Ins (almost 4 times larger than the right one), even when in the right hemisphere OFA/FFA is the larger target (1.5 larger), which could compensate to some extent the contribution from left Ins. Only one previous report found the left hemisphere connectivity lateralization, but it is related to STS [25] which agrees with our results. It’s important to notice that left laterization of connectivity was found in this study despite of the similar size of both STS, left and right.

In general, the left lateralization was found in the main sample when the tractography was ran and also in its replication in the HCP data with ROIs from second-level analysis while the use of individual ROIs (first-level analysis) resulted in no hemispheric difference pointing to the importance of ROIs selection for the tracking related to functional response. It can be possible that the size of the seeding ROIs affects the results of the tractography since the absolute connectivity value depends on the number of voxels in the seed region and the number of times each voxel is seeded for tractography [36, 45], explaining the left lateralization found here. Besides, the high degree of variability in ROIs coming from individual analysis [46, 47] seems to be a not suitable choice for generalization, at least in the network studied here.

In front of this unexpected left lateralization, the tractography was run again (on HCP data) but this time flipping the ROIs along the left-right axis. Interestingly, with the flipped ROIs the resulting probability maps presented a right lateralization of density of visits along the AAL structures, but in lesser extended that when the ROIs were seeded in their original position. These results showed that the seeding in flipped ROIs achieved only a 24.14% of right lateralization and 10.34% of left one, while the original run had a 32.76% of left lateralization and 0.86% of right one (for further details refers to Table in S1 Table). The effect of flipped ROIs here is not as strong as we were expecting in case of ROI size as the main cause for the lateralization. However, it is not possible to conclude about the left lateralization under these conditions, even when there is a tendency to stronger connections in the left hemisphere. This effect emphasizes the importance of reporting the actual size of the ROIs used in probabilistic tractography, which is not the common practice in previous reports.

Through this study, the identification of the core system of face processing was possible with the selected stimulation paradigm, which elicited larger activations clusters in the right hemisphere (Table 1), at least for the core regions. This lateralized activation to the right in face tasks is a widely reported fact that supports the hypothesis of right-hemisphere dominance for face-specific processing [5, 48]. However, the current models of familiar face recognition do not take into account the differences in processing between hemispheres where the right one seems to be related to high level individualization and categorization of persons and the left one is involved on mechanism of reshaping and restructuring the features in a appropriated context using verbally acquired abstract criteria [49]. This functional lateralization of hemispheres was tested using voxel-based morphometry and correlating performance in three tasks with whole-brain gray matter volume, finding significant correlation between performance in naming and semantic association with left anterior temporal lobe, whereas familiarity judgment with right anterior middle temporal gyrus [50].

The location of OFA/FFA has been very consistent in several studies [2, 5, 14, 51–53]. Specifically, the FFA has been identified as an area especially sensible to facial configuration and identity while OFA has been related to more basics characteristic of the faces [54, 55]. In addition to these areas, there is a third one located in the posterior part of the superior temporal sulcus (STSp), evinced in this study, that has been previously reported to be related to the perception of facial movements and in the perception of features, such as eye gaze and expression [6], as well as audiovisual face-voice integration region [56]. These areas that belong to the core face system presented activations that tended to be more extended in the right hemisphere which agrees with previous reports [5, 14] but in less extend in the STSp. It is important to note that the contrast used here for localizing activation areas is not proper for uncovering visual areas in a wide criterion, and the ROI representing visual area here is small and displaced from the calcarine cortex. That means we were extracting only the voxels that were responding preferentially to faces if we consider that Vis responds to a single object category [57]. This restriction in the number of voxels in the area called Vis, instead of use of a predefined ROIs from anatomical information, could explain the lack of connections found between visual areas and the face core system.

By using familiar faces as stimuli as well as the contrast familiar faces > unfamiliar faces, it was possible to successfully identify the areas corresponding to the extended system of face processing: mOF/AC, medial and posterior cingulate cortex (MC, PC) and Ins. Most of these areas were composed of more voxels in the left hemisphere (except for mOF/AC). It is important to note that this study used personally-familiar faces instead of famous or learned faces, which must enhance the activation of extended system and recruited areas related to processing the emotional meaning (faces from friends and relatives) of the faces, a subsystem related to the emotion-from identity processing [58] which is not activated in presence of visually familiar faces (learnt in laboratory). On the other hand, an interesting study using multivoxel pattern analysis to fMRI was able to decode identity-independent familiarity information and face identity in a set of overlapping areas in core and extended systems providing evidence that activity in the extended system might also carry information about identity and personal familiarity [59].

The larger activation of extended system areas in the left hemisphere in response to familiar faces can be explained by the lateralization of positive emotions to the left hemisphere that has been proposed based on the ‘valence hypothesis’ which assumes an opposite dominance of the left hemisphere for positive emotions and of the right hemisphere for negative emotions [60, 61]. Personally familiar faces elicited bilateral activation with important recruitment of the left hemisphere [58]. Leibenluft et al. (2004) by contrasting the face of a mother's own child and a familiar child, which found areas in the anterior paracingulate, pre-frontal cortex, and left insula which was considered like a maternal attachment sign [62]. Another study that distinguished between famous faces and personally familiar faces found that famous ones were right lateralized whereas personally-familiar faces, particularly partner and own faces, elicited bilateral activations [63], which agrees with our findings despite the lateralization for some ROIs.

The connectivity found between the core area and insula supports the existence of a direct white-matter route from occipital cortex to limbic areas bypassing other areas, like PC, which has been proposed referring to the amygdala [1, 25]. Despite the relatively low connectivity compared with other seed-target pairs, the presence of this connection among so far away regions and taking into account the limitation of the probabilistic tracking which values fall down with the distance [64], leads us to solidify the existence of connections among areas. Even when the tractography was not seeded in the amygdala (since it did not emerge from fMRI study) the trajectory of the high connectivity voxels from occipitotemporal to frontal areas, seems to bypass the amygdala (see Fig 3).

Ethofer et al. (2013) used an fMRI adaptation paradigm for faces and voices to determine regions to be used as seed in probabilistic tractography. They found that STS modules converge in the orbitofrontal cortex, which runs through the external capsule for the voice area, through the dorsal superior longitudinal fasciculus (SLF) for the face area and through the ventral SLF for the audiovisual integration area. However, we did not detect this pattern for STSp, perhaps due to methodological differences since they performed probabilistic fiber tracking without restrictions by the target.

On the other hand, Gschwind et al. (2012) found no preferential link from STS to FFA or OFA [3] what disagrees with our findings and contradicts the current cognitive models. Also, this ROI has shown the tendency to be connected with more frontal areas [25], in our case is evident the preferential connection to cingulate (medial and posterior) cortexes. The lack of strong connections found by Pyles et al. (2013) between STS and middle fusiform (referring as FFA in this article), or between STS and the other face-selective regions is the reason why this author suggested a re-evaluation of the “core” face network with respect to what functional areas are or are not included in this network. However, we did find relatively high connectivity toward more frontal areas.

Finally, it must be considered the pattern of activation found in the extended system and its possible role in familiar face processing information. Although the PC had presented low probability of direct connections to all other face-responsive areas for unfamiliar face [25], the present study found that this region, when is delimited by familiar faces, appears to some extend to all of them, having the high probability of connections towards MC, left STS and Vis. This area has been related to the assessment of personal relevance [65], face self-recognition [66], memory process, familiarity and affective valence [1], but the actual role is still unclear.

The mOF/AC was poorly connected to the other studied areas. This was unexpected since it is believe to be involved in the perception of emotional information in faces and voices and constitutes a neural interface linking sensory areas with brain regions implicated in the generation of behavioral responses [56]. This area, that belongs to the prefrontal cortex region did not respond to familiar faces lacking of semantic information, suggesting therefore, that this area is only modulated by personal knowledge triggered by familiar faces [67, 68]. In this study, the connectivity estimations seem to be affected by the lack of distance correction.

According to our data, the left insula response to the presented stimuli is more extended than its counterpart in the right hemisphere. In literature, the Ins responds to stimuli valence, such as facial expression [69] and as a mediator in empathic reactions [70]. The face of one's child elicits activity in this area reflecting the attachment and protectiveness in the maternal care [62]. A previous study from our group in Capgras syndrome showed that there is a lack of connectivity among occipital areas of face processing and frontal areas in left hemisphere associated to lack of affective processing of familiar faces [15] supporting the relevance of this hemisphere for emotional valence of face identity processing.

The cingulate regions belonging to the extended system of faces are related in general to emotional processing. MC has been associated to the facilitation of emotional face recognition where recognition of disgusted faces was improved by the presentation of an olfactory stimulus irrespective of its emotional valence [9] and self-awareness [71]. PC showed an increased activity when familiar faces (actors, politicians and family members) are presented suggesting that this region plays a role in the acquisition of simple visual familiarity [67, 72]; however, the response is stronger when the face belongs to a close acquaintances which might indicate the involvement of these areas in the retrieval of episodic memories and biographical information associated with familiar individuals [13]. Gschwind et al. (2012) in a connectivity analysis of posterior cingulate showed a low probability of connections to the other analyzed face-responsive areas what agrees in general with our result in which the main connections to this area were from Vis and MC.

### Limitations

Due to our interest in exploring the connectivity toward the regions that respond to familiarity in face processing (extended system), we analyzed two regions that belong to the core system as a unique region (OFA/FFA) in our main sample, which neglect some internal connection between the inferior occipital gyrus and middle fusiform gyrus and from these regions to the other explored ROIs. However, the probabilistic tractography using independent OFA and FFA on HCP dataset allowed to shed some light over possible neglected information. Other limitation of this study is that we analyzed the extended system regions, (mOF/AC, MC and PC) as unique ROIs without considering the hemispheric representation in each one. Besides what is mentioned above, it must take into consideration one of the limitations of the probabilistic tractography, namely the decreasing of probability with distance what could lead to an overestimation in the closer paths to the seed and an underestimation of real path towards more distant regions [64]. On the other hand, we performed group analysis to extract the ROIs which implies that only the face-selective voxels that overlap across individuals will be included, and thus many voxels in each individual are outside the region of overlap and not further analyzed [73].

Another important element that must be considered in this study is the handedness of participants. Interestingly, in a recent study the handedness has been related to neural mechanism underlying hemispheric lateralization of face processing [74]. These authors found an enhanced recruitment of the left FFA in left-handers compared to right-handers, while OFA was similarly lateralized to the right hemisphere in both groups. Also, they found that the gray matter volume in left FFA was significantly larger in left-handers. Another evidence was observed in prosopagnosia following unilateral lesions to the left hemisphere, which has been reported in left-handed subjects [75, 76]. Since our sample present heterogeneous handedness some of these effects could be included in the group analysis, despite of the found larger activation in the right hemisphere, at least for the core structures. After a recent report about the handedness effect on lateralization of face processing [74] this sample characteristic should be maintained homogenous across the subjects in our future work.

## Conclusions

The connectivity profile associated to familiar face processing circuitry seems to be based on parallel links between core and extended system of face areas, including direct anatomical connections between OFA/FFA and STS, and also direct connections also between OFA/FFA and insula. This direct occipito-frontal route could explain the emotional information processing related to familiar faces.

## Supporting information

S1 Fig Coincidence map of subject’s connectivity from the probabilistic tractography. A) Subsample recorded in Siemens scanner, B) in General Electric scanner. The map was created by including the voxels with more than 5000 visits.
(TIF)

S2 Fig Connectivity path among the individual functional seeds obtained from the first-level analysis of fMRI across nine subjects. The average of count of visits is drawn in each voxel. Color indicates the number of visits.
(TIF)

S1 Table Statistical report of hemispheric differences based on density of count of visit in AAL structures for the performed tractographies. (PDF)

## References

[1] Gobbini MI, Haxby JV. Neural systems for recognition of familiar faces. Neuropsychologia. 2007;45(1):32–41. 10.1016/j.neuropsychologia.2006.04.015 16797608

[2] Haxby JV, Hoffman EA, Gobbini MI. Human neural systems for face recognition and social communication. Biological psychiatry. 2002;51(1):59–67. 10.1016/s0006-3223(01)01330-0 11801231

[3] Pyles JA, Verstynen TD, Schneider W, Tarr MJ. Explicating the face perception network with white matter connectivity. PloS one. 2013;8(4):e61611 10.1371/journal.pone.0061611 23630602 PMC3632522

[4] Kanwisher N, Yovel G. The fusiform face area: a cortical region specialized for the perception of faces. Philosophical Transactions of the Royal Society B: Biological Sciences. 2006;361(1476):2109–28. 10.1098/rstb.2006.1934 17118927 PMC1857737

[5] Kanwisher N, McDermott J, Chun MM. The fusiform face area: a module in human extrastriate cortex specialized for face perception. The Journal of Neuroscience. 1997;17(11):4302–11. 9151747 10.1523/JNEUROSCI.17-11-04302.1997 PMC6573547

[6] Haxby JV, Hoffman EA, Gobbini MI. The distributed human neural system for face perception. Trends in cognitive sciences. 2000;4(6):223–33. 10827445 10.1016/s1364-6613(00)01482-0

[7] Ishai A, Schmidt CF, Boesiger P. Face perception is mediated by a distributed cortical network. Brain research bulletin. 2005;67(1):87–93. 16140166 10.1016/j.brainresbull.2005.05.027

[8] Bernard FA, Bullmore ET, Graham KS, Thompson SA, Hodges JR, Fletcher PC. The hippocampal region is involved in successful recognition of both remote and recent famous faces. Neuroimage. 2004;22(4):1704–14. 10.1016/j.neuroimage.2004.03.036 15275926

[9] Seubert J, Kellermann T, Loughead J, Boers F, Brensinger C, Schneider F, et al Processing of disgusted faces is facilitated by odor primes: a functional MRI study. Neuroimage. 2010;53(2):746–56. 10.1016/j.neuroimage.2010.07.012 20627130

[10] Breiter HC, Etcoff NL, Whalen PJ, Kennedy WA, Rauch SL, Buckner RL, et al Response and habituation of the human amygdala during visual processing of facial expression. Neuron. 1996;17(5):875–87. 10.1016/s0896-6273(00)80219-6 8938120

[11] Fairhall SL, Ishai A. Effective connectivity within the distributed cortical network for face perception. Cerebral cortex. 2007;17(10):2400–6. 10.1093/cercor/bhl148 17190969

[12] Ishai A. Let’s face it: it’sa cortical network. Neuroimage. 2008;40(2):415–9. 10.1016/j.neuroimage.2007.10.040 18063389

[13] Haxby JV, Gobbini MI. Distributed Neural Systems for Face Perception. Oxford Handbook of Face Perception. 2011:93.

[14] Haxby JV, Horwitz B, Ungerleider LG, Maisog JM, Pietrini P, Grady CL. The functional organization of human extrastriate cortex: a PET-rCBF study of selective attention to faces and locations. The Journal of Neuroscience. 1994;14(11):6336–53. 7965040 10.1523/JNEUROSCI.14-11-06336.1994 PMC6577268

[15] Bobes MA, Góngora D, Valdes A, Santos Y, Acosta Y, Fernandez-Garcia Y, et al Testing the connections within face processing circuitry in Capgras delusion with diffusion imaging tractography. NeuroImage: Clinical. 2016. 10.1016/j.nicl.2016.01.006 PMC4732187 26909325

[16] Góngora D, Iglesias-Fuster J, Martínez Y, Estevez N, Bobes M. Age-related Changes in White Matter Tracts Associated with Face Recognition System. Revista Neuropsicología, Neuropsiquiatría y Neurociencias. 2016;16(3):35–52.

[17] Thomas C, Moya L, Avidan G, Humphreys K, Jung KJ, Peterson MA, et al Reduction in white matter connectivity, revealed by diffusion tensor imaging, may account for age-related changes in face perception. Journal of Cognitive Neuroscience. 2008;20(2):268–84. 10.1162/jocn.2008.20025 18275334 PMC5733143

[18] Mori S, Wakana S, Van Zijl PC, Nagae-Poetscher L. MRI atlas of human white matter: Elsevier; 2005. 10.1148/radiol.2301021640 14645885

[19] Crosby EC. Correlative anatomy of the nervous system: Macmillan; 1962.

[20] Gloor P. The temporal lobe and limbic system: Oxford University Press, USA; 1997.

[21] Catani M, Howard RJ, Pajevic S, Jones DK. Virtual in vivo interactive dissection of white matter fasciculi in the human brain. Neuroimage. 2002;17(1):77–94. 12482069 10.1006/nimg.2002.1136

[22] Dejerine JJ. Anatomie des centres nerveux: Rueff; 1895.

[23] Behrens TE, Woolrich MW, Jenkinson M, Johansen‐Berg H, Nunes RG, Clare S, et al Characterization and propagation of uncertainty in diffusion‐weighted MR imaging. Magnetic resonance in medicine. 2003;50(5):1077–88. 10.1002/mrm.10609 14587019

[24] Yo T-S, Anwander A, KnAusche T, editors. Fiber cup 2009: Reconstructing fibers from the phantom data MICCAI workshop on Diffusion Modelling and the Fiber Cup (DMFC’09), London, United Kingdom; 2009.

[25] Gschwind M, Pourtois G, Schwartz S, Van De Ville D, Vuilleumier P. White-matter connectivity between face-responsive regions in the human brain. Cerebral cortex. 2012;22(7):1564–76. 10.1093/cercor/bhr226 21893680

[26] Weiner KS, Grill-Spector K. The improbable simplicity of the fusiform face area. Trends in cognitive sciences. 2012;16(5):251–4. 10.1016/j.tics.2012.03.003 22481071

[27] W.M. O. Declaration of Helsinki (1964). BMJ. 1996;(313):1448–9.8664621

[28] Van Essen DC, Smith SM, Barch DM, Behrens TE, Yacoub E, Ugurbil K, et al The WU-Minn human connectome project: an overview. 2013;80:62–79. 10.1016/j.neuroimage.2013.05.041 23684880 PMC3724347

[29] Friston KJ, Ashburner J, Kiebel SJ, Nichols T, Penny W. Statistical Parametric Mapping. 2007.

[30] Diedrichsen J, Shadmehr R. Detecting and adjusting for artifacts in fMRI time series data. Neuroimage. 2005;27(3):624–34. 10.1016/j.neuroimage.2005.04.039 15975828 PMC1479857

[31] Brett M, Anton J, Valabregue R, Pioline J. Region of interest analysis using an SPM toolbox [abstract] Presented at the 8th Internation Conference on Functional Mapping of the Human Brain,. Available on CD-ROM in NeuroImage. NeuroImage. 2002;16.

[32] The MathWorks I. MATLAB 2014a. 2014.

[33] Collignon A, Maes F, Delaere D, Vandermeulen D, Suetens P, Marchal G, editors. Automated multi-modality image registration based on information theory. Information processing in medical imaging; 1995.

[34] Tzourio-Mazoyer N, Landeau B, Papathanassiou D, Crivello F, Etard O, Delcroix N, et al Automated anatomical labeling of activations in SPM using a macroscopic anatomical parcellation of the MNI MRI single-subject brain. 2002;15(1):273–89. 10.1006/nimg.2001.0978 11771995

[35] Smith SM, Jenkinson M, Woolrich MW, Beckmann CF, Behrens TE, Johansen-Berg H, et al Advances in functional and structural MR image analysis and implementation as FSL. Neuroimage. 2004;23:S208–S19. 10.1016/j.neuroimage.2004.07.051 15501092

[36] Woolrich MW, Jbabdi S, Patenaude B, Chappell M, Makni S, Behrens T, et al Bayesian analysis of neuroimaging data in FSL. Neuroimage. 2009;45(1):S173–S86. 19059349 10.1016/j.neuroimage.2008.10.055

[37] Jenkinson M, Beckmann CF, Behrens TE, Woolrich MW, Smith SM. Fsl. Neuroimage. 2012;62(2):782–90. 10.1016/j.neuroimage.2011.09.015 21979382

[38] Smith SM. Fast robust automated brain extraction. Human brain mapping. 2002;17(3):143–55. 10.1002/hbm.10062 12391568 PMC6871816

[39] Behrens TE, Berg HJ, Jbabdi S, Rushworth MF, Woolrich MW. Probabilistic diffusion tractography with multiple fibre orientations: What can we gain? Neuroimage. 2007;34(1):144–55. 10.1016/j.neuroimage.2006.09.018 17070705 PMC7116582

[40] Groppe DM, Urbach TP, Kutas M. Mass univariate analysis of event‐related brain potentials/fields II: Simulation studies. Psychophysiology. 2011;48(12):1726–37. 10.1111/j.1469-8986.2011.01272.x 21895684 PMC4059014

[41] Groppe DM, Urbach TP, Kutas M. Mass univariate analysis of event‐related brain potentials/fields I: A critical tutorial review. Psychophysiology. 2011;48(12):1711–25. 10.1111/j.1469-8986.2011.01273.x 21895683 PMC4060794

[42] StatSoft I. STATISTICA (data analysis software system), version 10. 2011.

[43] Zalesky A, Fornito A, Harding IH, Cocchi L, Yücel M, Pantelis C, et al Whole-brain anatomical networks: does the choice of nodes matter? Neuroimage. 2010;50(3):970–83. 10.1016/j.neuroimage.2009.12.027 20035887

[44] Lazar M. Mapping brain anatomical connectivity using white matter tractography. NMR in Biomedicine. 2010;23(7):821–35. 10.1002/nbm.1579 20886567 PMC4503207

[45] Calabrese E, Badea A, Cofer G, Qi Y, Johnson GA. A diffusion MRI tractography connectome of the mouse brain and comparison with neuronal tracer data. Cerebral cortex. 2015;25(11):4628–37. 10.1093/cercor/bhv121 26048951 PMC4715247

[46] Davis T, LaRocque KF, Mumford JA, Norman KA, Wagner AD, Poldrack RAJN. What do differences between multi-voxel and univariate analysis mean? How subject-, voxel-, and trial-level variance impact fMRI analysis. 2014;97:271–83. 10.1016/j.neuroimage.2014.04.037 PMC4115449 24768930

[47] Thirion B, Pinel P, Tucholka A, Roche A, Ciuciu P, Mangin J-F, et al Structural analysis of fMRI data revisited: improving the sensitivity and reliability of fMRI group studies. J IEEE Transactions on Medical Imaging. 2007;26(9):1256–69. 10.1109/TMI.2007.903226 17896597

[48] Rossion B, Caldara R, Seghier M, Schuller AM, Lazeyras F, Mayer E. A network of occipito‐temporal face‐sensitive areas besides the right middle fusiform gyrus is necessary for normal face processing. Brain. 2003;126(11):2381–95. 12876150 10.1093/brain/awg241

[49] Gainotti G. Implications of recent findings for current cognitive models of familiar people recognition. J Neuropsychologia. 2015;77:279–87. 10.1016/j.neuropsychologia.2015.09.002 26359717

[50] Borghesani V, Narvid J, Battistella G, Shwe W, Watson C, Binney RJ, et al “Looks familiar, but I do not know who she is”: The role of the anterior right temporal lobe in famous face recognition. 2019;115:72–85. 10.1016/j.cortex.2019.01.006 PMC6759326 30772608

[51] Chao LL, Martin A, Haxby JV. Are face‐responsive regions selective only for faces? Neuroreport. 1999;10(14):2945–50. 10.1097/00001756-199909290-00013 10549802

[52] Halgren E, Dale AM, Sereno MI, Tootell RB, Marinkovic K, Rosen BR. Location of human face-selective cortex with respect to retinotopic areas. Human brain mapping. 1999;7(1):29–37. 9882088 10.1002/(SICI)1097-0193(1999)7:1<29::AID-HBM3>3.0.CO;2-R PMC6873292

[53] Osher DE, Saxe RR, Koldewyn K, Gabrieli JD, Kanwisher N, Saygin ZM. Structural connectivity fingerprints predict cortical selectivity for multiple visual categories across cortex. Cerebral Cortex. 2015:bhu303. 10.1093/cercor/bhu303 PMC4785945 25628345

[54] Rotshtein P, Henson RN, Treves A, Driver J, Dolan RJ. Morphing Marilyn into Maggie dissociates physical and identity face representations in the brain. Nature neuroscience. 2005;8(1):107–13. 10.1038/nn1370 15592463

[55] Liu J, Harris A, Kanwisher N. Perception of face parts and face configurations: an fMRI study. Journal of Cognitive Neuroscience. 2010;22(1):203–11. 10.1162/jocn.2009.21203 19302006 PMC2888696

[56] Ethofer T, Bretscher J, Wiethoff S, Bisch J, Schlipf S, Wildgruber D, et al Functional responses and structural connections of cortical areas for processing faces and voices in the superior temporal sulcus. Neuroimage. 2013;76:45–56. 10.1016/j.neuroimage.2013.02.064 23507387

[57] Haxby JV, Gobbini MI, Furey ML, Ishai A, Schouten JL, Pietrini P. Distributed and overlapping representations of faces and objects in ventral temporal cortex. Science. 2001;293(5539):2425–30. 10.1126/science.1063736 11577229

[58] Bobes MA, Castellanos AL, Quiñones I, García L, Valdes-Sosa M. Timing and tuning for familiarity of cortical responses to faces. PloS one. 2013;8(10):e76100 10.1371/journal.pone.0076100 24130761 PMC3794035

[59] di Oleggio Castello MV, Halchenko YO, Guntupalli JS, Gors JD, Gobbini MI. The neural representation of personally familiar and unfamiliar faces in the distributed system for face perception. Scientific reports. 2017;7(1):12237 10.1038/s41598-017-12559-1 28947835 PMC5612994

[60] Alves NT, Fukusima SS, Aznar-Casanova JAJP, Neuroscience. Models of brain asymmetry in emotional processing. 2008;1(1):63–6.

[61] Gainotti GJN. Emotions, unconscious processes, and the right hemisphere. 2005;7(1):71–81.

[62] Leibenluft E, Gobbini MI, Harrison T, Haxby JVJBp. Mothers' neural activation in response to pictures of their children and other children. 2004;56(4):225–32. 10.1016/j.biopsych.2004.05.017 15312809

[63] Taylor MJ, Arsalidou M, Bayless SJ, Morris D, Evans JW, Barbeau EJJHbm. Neural correlates of personally familiar faces: parents, partner and own faces. 2009;30(7):2008–20. 10.1002/hbm.20646 18726910 PMC6870744

[64] Morris DM, Embleton KV, Parker GJ. Probabilistic fibre tracking: differentiation of connections from chance events. Neuroimage. 2008;42(4):1329–39. 10.1016/j.neuroimage.2008.06.012 18619548

[65] Vogt BA, Vogt L, Laureys S. Cytology and functionally correlated circuits of human posterior cingulate areas. Neuroimage. 2006;29(2):452–66. 10.1016/j.neuroimage.2005.07.048 16140550 PMC2649771

[66] Platek SM, Loughead JW, Gur RC, Busch S, Ruparel K, Phend N, et al Neural substrates for functionally discriminating self‐face from personally familiar faces. Human brain mapping. 2006;27(2):91–8. 10.1002/hbm.20168 16035037 PMC6871291

[67] Gobbini MI, Haxby JV. Neural response to the visual familiarity of faces. Brain research bulletin. 2006;71(1):76–82. 17113931 10.1016/j.brainresbull.2006.08.003

[68] Mitchell JP, Heatherton TF, Macrae CN. Distinct neural systems subserve person and object knowledge. Proceedings of the National Academy of Sciences. 2002;99(23):15238–43. 10.1073/pnas.232395699 PMC137574 12417766

[69] Phillips ML, Drevets WC, Rauch SL, Lane R. Neurobiology of emotion perception I: The neural basis of normal emotion perception. Biological psychiatry. 2003;54(5):504–14. 10.1016/s0006-3223(03)00168-9 12946879

[70] Singer T, Seymour B, O'doherty J, Kaube H, Dolan RJ, Frith CD. Empathy for pain involves the affective but not sensory components of pain. Science. 2004;303(5661):1157–62. 10.1126/science.1093535 14976305

[71] Posner MI, Rothbart MK. Attention, self–regulation and consciousness. Philosophical Transactions of the Royal Society B: Biological Sciences. 1998;353(1377):1915–27. 10.1098/rstb.1998.0344 PMC1692414 9854264

[72] Kosaka H, Omori M, Iidaka T, Murata T, Shimoyama T, Okada T, et al Neural substrates participating in acquisition of facial familiarity: an fMRI study. Neuroimage. 2003;20(3):1734–42. 14642483 10.1016/s1053-8119(03)00447-6

[73] Heller R, Golland Y, Malach R, Benjamini Y. Conjunction group analysis: an alternative to mixed/random effect analysis. Neuroimage. 2007;37(4):1178–85. 10.1016/j.neuroimage.2007.05.051 17689266

[74] Frässle S, Krach S, Paulus FM, Jansen A. Handedness is related to neural mechanisms underlying hemispheric lateralization of face processing. Scientific reports. 2016;6:27153 10.1038/srep27153 27250879 PMC4890016

[75] Barton JJ. Prosopagnosia associated with a left occipitotemporal lesion. Neuropsychologia. 2008;46(8):2214–24. 10.1016/j.neuropsychologia.2008.02.014 18374372

[76] Mattson AJ, Levin HS, Grafman J. A case of prosopagnosia following moderate closed head injury with left hemisphere focal lesion. Cortex. 2000;36(1):125–37. 10728902 10.1016/s0010-9452(08)70841-4

