# Supplementary figures and images for: Anatomical connections underlying personally-familiar face processing
Source: PLoS One. 2019 Sep 11;14(9):e0222087. doi: 10.1371/journal.pone.0222087 (PMC6738923; doi:10.1371/journal.pone.0222087)

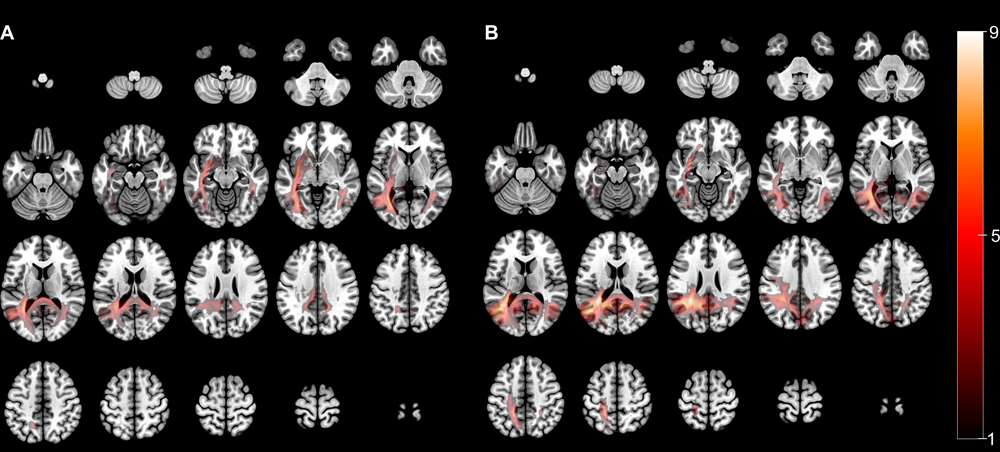

Supplement: S1 Fig — A) Subsample recorded in Siemens scanner, B) in General Electric scanner. The map was created by including the voxels with more than 5000 visits. (TIF) [file pone.0222087.s001.tif]

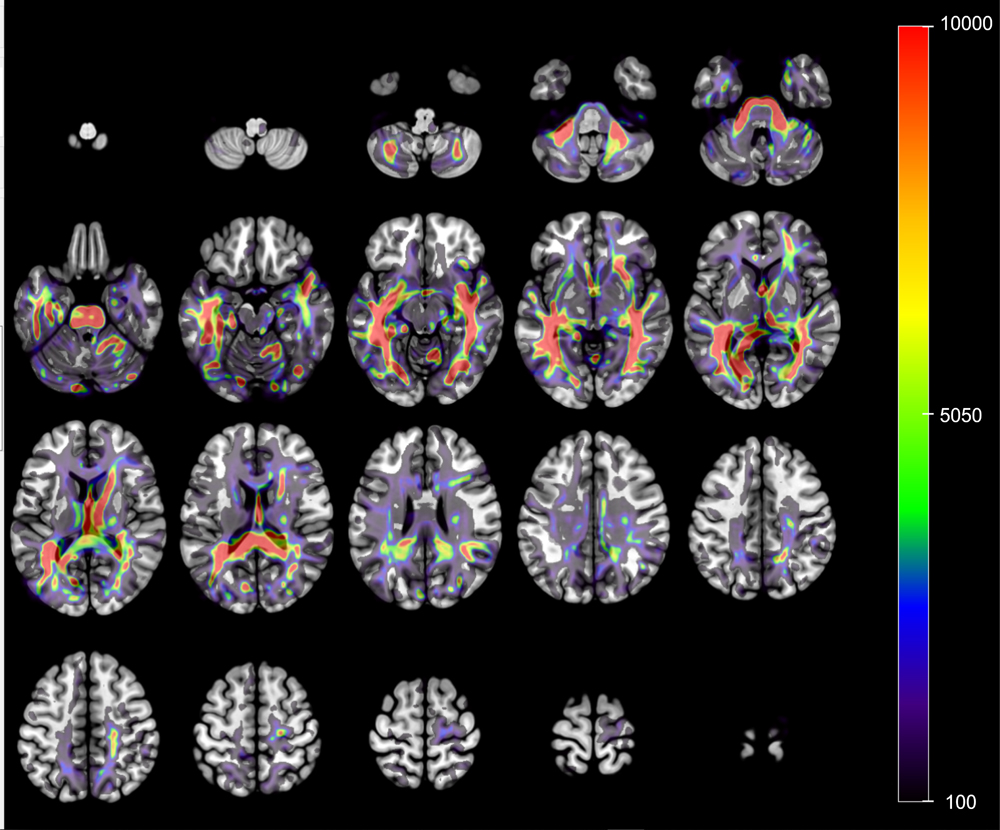

Supplement: S2 Fig — The average of count of visits is drawn in each voxel. Color indicates the number of visits. (TIF) [file pone.0222087.s002.tif]
