# Supplementary material for: Anatomical connections underlying personally-familiar face processing
Source: PLoS One. 2019 Sep 11;14(9):e0222087. doi: 10.1371/journal.pone.0222087 (PMC6738923; doi:10.1371/journal.pone.0222087)
Supplement: S1 Table — (PDF) [file pone.0222087.s003.pdf]

AAL labels/Dataset	Tracking using ROIs from second-level analysis fMRI		Tracking using ROIs from second-level analysis fMRI in HCP data		Tracking using ROIs from first-level analysis fMRI		Tracking using ROIs from second-level analysis fMRI in HCP data flipped along the left-right axis	
	pval	t-val	pval	t-val	pval	t-val	pval	t-val
Precentral_L	0.002	3.033	0.000	6.464	0.702	-0.542	1.000	-5.765
Frontal_Sup_L	0.264	0.639	0.007	2.571	0.858	-1.110	1.000	-4.023
Frontal_Sup_Orb_L	0.026	2.023	0.000	3.975	0.642	-0.371	0.935	-1.550
Frontal_Mid_L	0.001	3.237	0.000	4.233	0.443	0.145	0.999	-3.496
Frontal_Mid_Orb_L	0.036	1.861	0.000	3.969	0.712	-0.572	0.973	-1.984
Frontal_Inf_Oper_L	0.422	0.198	0.473	0.068	0.659	-0.418	0.014	2.297
Frontal_Inf_Tri_L	0.083	1.415	0.110	1.249	0.718	-0.589	0.010	2.436
Frontal_Inf_Orb_L	0.015	2.264	0.000	5.472	0.741	-0.662	1.000	-4.065
Rolandic_Oper_L	0.020	2.132	0.244	0.702	0.182	0.936	0.812	-0.895
Supp_Motor_Area_L	0.476	0.060	0.003	2.931	0.878	-1.207	0.972	-1.968
Olfactory_L	0.002	3.131	0.000	3.618	0.264	0.644	0.996	-2.780
Frontal_Sup_Medial_L	0.461	0.098	0.180	0.926	0.351	0.389	0.001	3.357
Frontal_Med_Orb_L	0.031	1.923	0.031	1.926	0.523	-0.059	0.104	1.279
Rectus_L	0.001	3.392	0.001	3.530	0.411	0.229	0.844	-1.027
Insula_L	0.015	2.262	0.016	2.215	0.771	-0.760	0.996	-2.796
Cingulum_Ant_L	0.172	0.960	0.182	0.917	0.426	0.190	0.001	3.381
Cingulum_Mid_L	0.137	1.113	0.555	-0.138	0.748	-0.685	0.000	3.758
Cingulum_Post_L	0.007	2.603	0.031	1.920	0.316	0.488	0.003	2.915
Hippocampus_L	0.000	4.389	0.000	4.231	0.535	-0.090	1.000	-4.607
ParaHippocampal_L	0.011	2.385	0.053	1.657	0.344	0.408	0.398	0.261
Amygdala_L	0.001	3.476	0.000	4.879	0.535	-0.090	1.000	-6.609
Calcarine_L	0.506	-0.015	0.835	-0.986	0.516	-0.042	1.000	-4.661
Cuneus_L	0.315	0.485	0.967	-1.892	0.245	0.708	0.074	1.477
Lingual_L	0.039	1.820	0.000	4.290	0.659	-0.417	1.000	-4.195
Occipital_Sup_L	0.048	1.717	0.663	-0.424	0.386	0.295	0.915	-1.401
Occipital_Mid_L	0.000	5.430	0.000	13.033	0.180	0.943	0.004	2.799
Occipital_Inf_L	0.013	2.315	0.000	8.643	0.556	-0.143	1.000	-5.127
Fusiform_L	0.000	4.045	0.000	4.812	0.221	0.787	0.048	1.703
Postcentral_L	0.002	3.092	0.000	4.757	0.804	-0.881	1.000	-5.446

Parietal_Sup_L	0.000	3.746	0.000	5.457	0.660	-0.420	1.000	-3.601
Parietal_Inf_L	0.002	3.125	0.000	5.211	0.659	-0.418	0.839	-1.005
SupraMarginal_L	0.001	3.380	0.000	3.891	0.914	-1.428	1.000	-5.231
Angular_L	0.008	2.515	0.000	7.251	0.775	-0.775	1.000	-8.000
Precuneus_L	0.198	0.860	0.672	-0.449	0.374	0.328	0.013	2.313
Paracentral_Lobule_L	0.007	2.586	0.066	1.542	0.802	-0.873	0.553	-0.135
Caudate_L	0.001	3.286	0.004	2.839	0.666	-0.437	0.965	-1.858
Putamen_L	0.000	3.773	0.000	6.456	0.776	-0.776	1.000	-9.315
Pallidum_L	0.000	5.413	0.002	2.996	0.814	-0.919	1.000	-6.128
Thalamus_L	0.000	5.531	0.000	3.823	0.755	-0.706	0.999	-3.257
Heschl_L	0.000	4.194	0.130	1.145	0.811	-0.905	0.951	-1.691
Temporal_Sup_L	0.237	0.724	0.427	0.186	0.724	-0.606	1.000	-4.023
Temporal_Pole_Sup_L	0.015	2.277	0.004	2.765	0.777	-0.783	0.985	-2.247
Temporal_Mid_L	0.000	5.424	0.000	21.141	0.293	0.555	1.000	-13.319
Temporal_Pole_Mid_L	0.622	-0.314	0.001	3.513	0.769	-0.753	1.000	-6.204
Temporal_Inf_L	0.087	1.390	0.000	3.653	0.143	1.105	1.000	-7.818
Cerebelum_Crus1_L	0.004	2.771	0.000	3.653	0.630	-0.337	0.050	1.689
Cerebelum_Crus2_L	0.026	2.018	0.005	2.746	0.489	0.028	0.816	-0.910
Cerebelum_3_L	0.089	1.377	0.016	2.225	0.832	-0.990	1.000	-5.038
Cerebelum_4_5_L	0.222	0.775	0.506	-0.016	0.809	-0.899	0.026	1.998
Cerebelum_6_L	0.041	1.792	0.072	1.493	0.869	-1.160	0.868	-1.136
Cerebelum_7b_L	0.073	1.484	0.002	3.112	0.241	0.720	0.088	1.380
Cerebelum_8_L	0.009	2.469	0.002	3.095	0.422	0.199	0.668	-0.439
Cerebelum_9_L	0.004	2.849	0.004	2.770	0.232	0.751	0.516	-0.040
Cerebelum_10_L	0.256	0.662	0.714	-0.569	0.509	-0.022	0.574	-0.188
Vermis_1_2	0.989	-2.395	0.852	-1.058	0.204	0.850	0.890	-1.245
Vermis_4_5	0.001	3.521	0.669	-0.440	0.160	1.024	0.850	-1.050
Vermis_7	0.011	2.411	0.000	4.366	0.174	0.966	0.004	2.784
Vermis_9	0.497	0.006	0.617	-0.299	0.564	-0.164	0.676	-0.461
Precentral_R	0.998	3.033	1.000	6.464	0.298	-0.542	0.000	-5.765
Frontal_Sup_R	0.736	0.639	0.993	2.571	0.142	-1.110	0.000	-4.023
Frontal_Sup_Orb_R	0.974	2.023	1.000	3.975	0.358	-0.371	0.065	-1.550
Frontal_Mid_R	0.999	3.237	1.000	4.233	0.557	0.145	0.001	-3.496
Frontal_Mid_Orb_R	0.964	1.861	1.000	3.969	0.288	-0.572	0.027	-1.984

Frontal_Inf_Oper_R	0.578	0.198	0.527	0.068	0.341	-0.418	0.986	2.297
Frontal_Inf_Tri_R	0.917	1.415	0.890	1.249	0.282	-0.589	0.990	2.436
Frontal_Inf_Orb_R	0.985	2.264	1.000	5.472	0.259	-0.662	0.000	-4.065
Rolandic_Oper_R	0.980	2.132	0.756	0.702	0.818	0.936	0.188	-0.895
Supp_Motor_Area_R	0.524	0.060	0.997	2.931	0.122	-1.207	0.028	-1.968
Olfactory_R	0.998	3.131	1.000	3.618	0.736	0.644	0.004	-2.780
Frontal_Sup_Medial_R	0.539	0.098	0.820	0.926	0.649	0.389	0.999	3.357
Frontal_Med_Orb_R	0.969	1.923	0.969	1.926	0.477	-0.059	0.896	1.279
Rectus_R	0.999	3.392	0.999	3.530	0.589	0.229	0.156	-1.027
Insula_R	0.985	2.262	0.984	2.215	0.229	-0.760	0.004	-2.796
Cingulum_Ant_R	0.828	0.960	0.818	0.917	0.574	0.190	0.999	3.381
Cingulum_Mid_R	0.863	1.113	0.445	-0.138	0.252	-0.685	1.000	3.758
Cingulum_Post_R	0.993	2.603	0.969	1.920	0.684	0.488	0.997	2.915
Hippocampus_R	1.000	4.389	1.000	4.231	0.465	-0.090	0.000	-4.607
ParaHippocampal_R	0.989	2.385	0.947	1.657	0.656	0.408	0.602	0.261
Amygdala_R	0.999	3.476	1.000	4.879	0.465	-0.090	0.000	-6.609
Calcarine_R	0.494	-0.015	0.165	-0.986	0.484	-0.042	0.000	-4.661
Cuneus_R	0.685	0.485	0.033	-1.892	0.755	0.708	0.926	1.477
Lingual_R	0.961	1.820	1.000	4.290	0.341	-0.417	0.000	-4.195
Occipital_Sup_R	0.952	1.717	0.337	-0.424	0.614	0.295	0.085	-1.401
Occipital_Mid_R	1.000	5.430	1.000	13.033	0.820	0.943	0.996	2.799
Occipital_Inf_R	0.987	2.315	1.000	8.643	0.444	-0.143	0.000	-5.127
Fusiform_R	1.000	4.045	1.000	4.812	0.779	0.787	0.952	1.703
Postcentral_R	0.998	3.092	1.000	4.757	0.196	-0.881	0.000	-5.446
Parietal_Sup_R	1.000	3.746	1.000	5.457	0.340	-0.420	0.000	-3.601
Parietal_Inf_R	0.998	3.125	1.000	5.211	0.341	-0.418	0.161	-1.005
SupraMarginal_R	0.999	3.380	1.000	3.891	0.086	-1.428	0.000	-5.231
Angular_R	0.992	2.515	1.000	7.251	0.225	-0.775	0.000	-8.000
Precuneus_R	0.802	0.860	0.328	-0.449	0.626	0.328	0.987	2.313
Paracentral_Lobule_R	0.993	2.586	0.934	1.542	0.198	-0.873	0.447	-0.135
Caudate_R	0.999	3.286	0.996	2.839	0.334	-0.437	0.035	-1.858
Putamen_R	1.000	3.773	1.000	6.456	0.224	-0.776	0.000	-9.315
Pallidum_R	1.000	5.413	0.998	2.996	0.186	-0.919	0.000	-6.128
Thalamus_R	1.000	5.531	1.000	3.823	0.245	-0.706	0.001	-3.257
Heschl_R	1.000	4.194	0.870	1.145	0.189	-0.905	0.049	-1.691

Temporal_Sup_R	0.763	0.724	0.573	0.186	0.276	-0.606	0.000	-4.023
Temporal_Pole_Sup_R	0.985	2.277	0.996	2.765	0.223	-0.783	0.015	-2.247
Temporal_Mid_R	1.000	5.424	1.000	21.141	0.707	0.555	0.000	-13.319
Temporal_Pole_Mid_R	0.378	-0.314	0.999	3.513	0.231	-0.753	0.000	-6.204
Temporal_Inf_R	0.913	1.390	1.000	3.653	0.857	1.105	0.000	-7.818
Cerebelum_Crus1_R	0.996	2.771	1.000	3.653	0.370	-0.337	0.950	1.689
Cerebelum_Crus2_R	0.974	2.018	0.995	2.746	0.511	0.028	0.184	-0.910
Cerebelum_3_R	0.911	1.377	0.984	2.225	0.168	-0.990	0.000	-5.038
Cerebelum_4_5_R	0.778	0.775	0.494	-0.016	0.191	-0.899	0.974	1.998
Cerebelum_6_R	0.959	1.792	0.928	1.493	0.131	-1.160	0.132	-1.136
Cerebelum_7b_R	0.927	1.484	0.998	3.112	0.759	0.720	0.912	1.380
Cerebelum_8_R	0.991	2.469	0.998	3.095	0.578	0.199	0.332	-0.439
Cerebelum_9_R	0.996	2.849	0.996	2.770	0.768	0.751	0.484	-0.040
Cerebelum_10_R	0.744	0.662	0.286	-0.569	0.491	-0.022	0.426	-0.188
Vermis_3	0.011	-2.395	0.148	-1.058	0.796	0.850	0.110	-1.245
Vermis_6	0.999	3.521	0.331	-0.440	0.840	1.024	0.150	-1.050
Vermis_8	0.989	2.411	1.000	4.366	0.826	0.966	0.996	2.784
Vermis_10	0.503	0.006	0.383	-0.299	0.436	-0.164	0.324	-0.461
